# Evaluation of the Safety and Toxicity of the Original Copper Nanocomposite Based on Poly-N-vinylimidazole

**DOI:** 10.3390/nano12010016

**Published:** 2021-12-22

**Authors:** Irina A. Shurygina, Galina F. Prozorova, Irina S. Trukhan, Svetlana A. Korzhova, Nataliya N. Dremina, Artem I. Emel’yanov, Olesya V. Say, Nadezhda P. Kuznetsova, Alexander S. Pozdnyakov, Michael G. Shurygin

**Affiliations:** 1 Irkutsk Scientific Center of Surgery and Traumatology, 1 Bortsov Revolutsii Street, 664003 Irkutsk, Russia; PREDEL4@yandex.ru (I.S.T.); drema76@mail.ru (N.N.D.); leechka1986@mail.ru (O.V.S.); mshurygin@gmail.com (M.G.S.); 2 A.E. Favorsky Irkutsk Institute of Chemistry, Siberian Branch, Russian Academy of Sciences, 1 Favorsky Street, 664033 Irkutsk, Russia; prozorova@irioch.irk.ru (G.F.P.); korzhova@irioch.irk.ru (S.A.K.); emelyanov@irioch.irk.ru (A.I.E.); nkuznetsova@irioch.irk.ru (N.P.K.); pozdnyakov@irioch.irk.ru (A.S.P.)

**Keywords:** copper nanocomposite, poly-N-vinylimidazole, cytotoxicity, hepatocyte, copper-dependent enzyme systems

## Abstract

A new original copper nanocomposite based on poly-N-vinylimidazole was synthesized and characterized by a complex of modern physicochemical and biological methods. The low cytotoxicity of the copper nanocomposite in relation to the cultured hepatocyte cells was found. The possibility to involve the copper from the nanocomposite in the functioning of the copper-dependent enzyme systems was evaluated during the incubation of the hepatocyte culture with this nanocomposite introduced to the nutrient medium. The synthesized new water-soluble copper-containing nanocomposite is promising for biotechnological and biomedical research as a new non-toxic hydrophilic preparation that is allowed to regulate the work of key enzymes involved in energy metabolism and antioxidant protection as well as potentially serving as an additional source of copper.

## 1. Introduction

Copper is one of the main trace elements necessary for the normal development of mammals. In natural systems, it can exist in both an oxidized (Cu^2+^) or reduced (Cu^+^) state. It is known that an adult consumes an average of 0.6 to 2 mg of copper per day with a total absorption of the element in the intestine at 20–50% (mostly in Cu^2+^ state), and the safe daily intake of this microelement for an adult is 10–12 mg [1,2,3,4]. The distribution of Cu in the organism occurs in two phases. The first phase involves the transport of ingested copper through the portal vein to the liver and further to the kidneys by binding to albumin and transcuprein. During the second phase, ceruloplasmin synthesized in the liver ensures the redistribution of copper to other tissues and organs, mainly to the heart, lungs and brain, binding approximately 60–90% of the circulating Cu in the blood in both the Cu^2+^ and Cu^+^ state. The excess of the trace element is transported back to the liver. Normally, the liver of an adult contains from 18 to 55 µg of copper per gram of dry weight, and, along with the brain and kidneys, is regarded as the organs containing the greatest amount of this element [3,4,5]. In addition, the liver provides the excretion of this trace element in the bile, which is the main way of copper excretion from the body. It accounts for up to 80% of the copper removed by the liver [2,4,6].

Due to the ability to exist in different redox states, copper ions can act as catalytic cofactors for various enzymes involved in such fundamental processes as oxidative phosphorylation, antioxidant protection, the formation of connective tissue, blood coagulation, iron transport, synthesis of neurotransmitters, and others [7]. In particular, copper is a cofactor for such key enzymes as superoxide dismutase, cytochrome c oxidase (CcO), and tyrosinase [8]. However, the most important function of copper is the one associated with energy metabolism and based on the activity of mitochondrial CcO, which is a heme-copper terminal oxidase playing the role of the final acceptor in the electron transfer chain and necessary for ATP production and molecular oxygen reduction [6,9].

Despite the importance of copper for the cell energy metabolism and antioxidant protection, excess amounts of metal ions are extremely toxic since they can serve as a source of reactive oxygen and nitrogen species (ROS and RNS, respectively), the generation of which leads to the development of oxidative stress accompanied by membrane lipid peroxidation, damage to proteins and nucleic acids, and finally results in cell death by apoptotic or necrotic pathways [6,10]. In this regard, under normal physiological conditions in the cellular content, the amount of this trace element in a free state is counted as less than one free ion per cell. This is explained by the fact that the predominant amount of copper in mammalian cells is present in a form associated with proteins or peptides notably—various metallothioneins, metallochaperones, and glutathione (GSH) [10,11,12]. At that, the literature notes the special significance of the latter for the free copper ion chelation within cells. Moreover, it was found that most of the copper ions in cells (up to 60%) are in the form associated with GSH [11].

It is known from medical practice that pathologies resulting from the accumulation in the body of excess copper amounts transcending the buffer capacity of cells are usually associated with disorders in liver functioning. This organ is considered to play the role of a Cu depot in the organism and, accordingly, carry the main toxic load when an unregulated increase in this element level occurs in the body [13,14,15,16]. Occupational hazards, environmental pollution or congenital metabolic disorders can lead to diseases or disturbances, among which Wilson–Konovalov disease and North American Indian childhood cirrhosis are the best studied. All of them are characterized by the accumulation of toxic copper concentrations in the liver (from 190 to 3360 µg/g dry weight) and associated damage to this organ of varying severity [5,13,17,18,19].

The dual significance of copper for human health induces the search for new low-toxic copper-containing compounds suitable for use in various fields of medicine and biotechnology. In this regard, the particular attention of researchers is attracted by such a rapidly developing area as the chemistry of nanocomposites containing copper in the form of nanosized particles. The application fields of such nanocomposites having a wide range of unique properties are quite diverse and include effective biomedical materials, inexpensive catalytic systems, optical nanosensors, and ecological biosorbents developing. Nevertheless, the usage of new copper-containing nanocomposites as an alternative to organic copper compounds in pharmacology and medicine is most promising [20]. Thus, it was shown that copper nanoparticles (CuNPs and CuO NPs) can be applied as antimicrobial, antifungal, anticancer, and anti-inflammatory agents as well as therapeutic agents for wound healing [8,21]. In this case, the antibacterial, antifungal and antitumor properties of these nanocomposites are caused by their cytotoxicity [22,23,24,25]. Despite the fact that the cytotoxicity of Cu/CuO NPs has been demonstrated in relation to many cancer cell lines [26] (e.g., to HepG2 breast cancer cells [27]; HeLa cell lines [28,29]; AMJ-13 breast cancer, SKOV-3 ovarian cancer [30]; lung cancer A549 [31,32]; prostate cancer PC-3 [33]), the mechanisms mediating these effects are currently not well understood. It has been suggested that CuNPs can release ions, induce intracellular ROS generation, or directly interact with cells [21]. In addition, it was shown that CuO NPs can injure the mitochondrion structure, which is accompanied by the loss of membrane potential and a decrease in respiratory activity in HeLa cervical cancer cells [28]. Conversely, studies have suggested that an increased ROS level detected in cancer cells is associated with their high metabolic activities.

At the same time, the toxicity of CuNPs against normal cells and vital human organs can cause undesired adverse effects. The data on the cytotoxicity of CuNPs differ to a significant extent and, apparently, depend on both the biological object under study and the nature of the analyzed nanocomposites. For example, for different cell lines, it was noted that IC50 ranged from 54.34 µg/mL [30] to 1000 µg/mL [34]; for animal models (Swiss albino mice), the lethal dose was 800 mg/kg [30]. However, plant-mediated CuNPs were demonstrated to be relatively safe for normal human cell lines [8].

Another important aspect that affects both the properties of CuNPs and the characteristics of the nanocomposite in whole is the choice of the matrix. During the formation of CuNPs, problems caused by the copper activity associated with aggregation and oxidation arise. A topical area of research is the targeted synthesis of nanoparticles of a certain size with a narrowly dispersed distribution and high stability of chemical and biological properties for a long time. Natural and synthetic polymers are promising for this purpose. An important role of polymers in the formation of metal nanoparticles lies in their ability to screen the resulting nanoparticles in the nanoscale state, prevent their aggregation, and ensure long-term stability of their properties. The effective uses of polymers as matrices stabilizing copper and copper oxide nanoparticles such as cellulose [35,36,37,38], chitosan [39,40], polystyrene, polyaniline, polybutylene, polyurethane, polyethylene glycol [41,42,43,44,45,46,47,48] have been reported. Another promising polymer for stabilizing nanoparticles is poly-N-vinylimidazole (PVI). The azole fragments of PVI macromolecules contain pyridine nitrogen atoms with electron-donating properties, which are capable of coordinating metal ions [49,50]. Along with this, PVI is readily soluble in water, has physiological activity, and is widely used in medicine [51,52].

In this work, we report on the synthesis of water-soluble PVI and its successful use in the formation of CuNPs. All the necessary modern research methods were used to study the structure and physicochemical properties of the polymer and the nanocomposite based on it. Particular attention is paid to the discussion of the nanocomposite biological activity as well as the research of its safety and the degree of toxicity. Using a primary culture of hepatocytes, the effect of a nanocomposite with CuNPs was compared with the one of organic salt of this metal regarding the CcO and glutathione synthetase activity in cells, as well as to the activity in the medium of enzymes reflecting destructive processes and used as markers of cytolysis.

## 2. Materials and Methods

### 2.1. Materials

N-vinylimidazole from Sigma-Aldrich (St. Louis, MO, USA) was used as monomer for the synthesis of polymers. α,α′-Azobisisobutyronitrile (AIBN) from Merck KGaA (Darmstadt, Germany) was utilized as a radical polymerization initiator. Copper acetate monohydrate (Cu(CH_3_COO)_2_∙H_2_O) and ascorbic acid (C_6_H_8_O_6_) from Sigma-Aldrich (St. Louis, MO, USA) were applied as a metallic precursor of CuNPs and the reducing agent, respectively, without further purification. Deionized water (H_2_O) was used to prepare solutions. Ethanol (C_2_H_5_OH) and acetone ((CH_3_)_2_CO) were distilled and purified according to the known procedures. Argon with a purity of 99.999 was utilized in all reactions.

Fetal bovine serum (FBS), collagenase, Hanks’ Balanced Salt (HBSS) were obtained from Sigma-Aldrich Co., (St. Louis, MO, USA). Williams’ Medium E, Dexamethasone and Cell Maintenance Coctail-B were acquired from Gibco (Thermo Fisher Scientific, Waltham, MA, USA). Anti-OxPhos Complex IV subunit I monoclonal antibody (Cat. ND 0589), Alexa fluor 568 goat anti-mouse IgG (H + L) (Cat. NA-11031), Alexa fluor 488 goat anti-rabbit IgG (H + L) (Cat. NA-11034), Hoechst (Cat. NH-3570) were purchased from Invitrogen (Carlsbad, CA, USA), Anti-Glutathione Synthetase antibody (Cat. ab133592) were acquired from Abcam (Cambridge, UK). Cell filter and 24-well plates were obtained from Corning (BioCoat, Horsham, PA, USA). We used heparin (Lek, Minsk, Belarus), Telazol (Zoetis Inc., Parsippany, NJ, USA), Chloroform (Ruiyuan Group Limited, Yingtan, China), set of reagents for determining the activity of ALT (Cat. B-8079) and AST-UV-Novo liquid form (Cat. B-8081) (Vector-Best, Novosibirsk, Russia), Alkaline phosphatase Kit, IFCC Modified Method (Mindray, Shenzhen, China).

### 2.2. Synthesis of Poly-N-vinylimidazole

Poly-N-vinylimidazole (PVI) was synthesized by the method of free radical polymerization of N-vinylimidazole (VI) using AIBN as an initiator. The reaction was carried out in an ethanol solution and argon atmosphere at a temperature of 70 °C. VI (3.8 g, 40.0 mmol) and AIBN (0.05 g, 0.3 mmol) were dissolved in 2 mL of ethanol and placed in a glass ampoule. The ampoule with the reaction mixture was filled with argon, sealed and kept in a thermostat at 70 °C for 24 h. The product formed at the end of the polymerization in the form of a transparent glassy block was dissolved in ethanol and precipitated with acetone. The polymer was filtered off and dried in vacuum over P_2_O_5_ at 50 °C until constant weight. The polymer was then dissolved in water, purified by dialysis for 48 h through a 5 kDa cellulose membrane (Cellu Sep H1, MFPI, Seguin, TX, USA) and freeze dried. As a result, the PVI polymer was obtained with a yield of 97% in the form of a white powder readily soluble in water, DMSO, DMF and DMAA.

### 2.3. Synthesis of CuNPs Nanocomposite

The synthesis of CuNPs nanocomposite (NC(Cu-PVI)) was carried out by the method of chemical reduction of copper ions from copper acetate monohydrate (CuAc_2_) under the action of an ascorbic acid agent in an aqueous solution of PVI polymer and argon atmosphere. PVI (1.0 g, 10.6 mmol) and CuAc_2_ (0.04 g, 0.2 mmol) were dissolved in 20 mL of water and stirred vigorously for 40 min at room temperature. Then the mixture was heated to 80 °C and an aqueous solution containing 0.37 g, 2.1 mmol of ascorbic acid was added dropwise over 5 min, and the reaction mixture was stirred continuously for 2 h at 80 °C. The resulting suspension was precipitated with acetone and filtered to isolate the nanocomposite. The nanocomposite was then purified by dialysis against water through a cellulose membrane (Cellu Sep H1, MFPI, Seguin, TX, USA) and freeze dried. As a result, a nanocomposite with CuNPs in a PVI matrix was obtained in the form of a dark brown powder readily soluble in water, DMSO, DMF, and DMAA. The copper content in the nanocomposite is 1.0%.

### 2.4. Instruments for Characterization of Polymer and NC(Cu-PVI)

The structure and properties of the synthesized PVI polymer and a CuNPs nanocomposite were studied using all the necessary modern methods: FTIR, UV, and NMR spectroscopy, gel permeation chromatography, elemental and atomic absorption analysis, electron microscopy, dynamic light scattering, and thermogravimetry. FTIR spectra were recorded on a Vertex 70 spectrometer (Bruker Corporation, Billerica, MA, USA) in the range of 550–4000 cm^−1^. UV–visible spectra were recorded on a UV–Vis spectrophotometer Lambda 35 (Perkin-Elmer, Waltham, MA, USA) in the range of 300–900 nm. The molecular weight of the polymer was determined using Shimadzu LC-20 Prominence system (Shimadzu Corporation, Kyoto, Japan) fitted with a differential refractive index detector Shimadzu RID-20A and column Agilent PolyPore 7.5 mm × 300 mm (PL1113-6500) (Agilent Technologies, Santa Clara, CA, USA). A solution of N,N-dimethylformamide was used as eluent at a flow rate of 1 mL/min. Samples were dissolved at 50 °C for 24 h with stirring. Calibration was performed using a series of Polystyrene High EasiVials polystyrene standards (PL2010-0201) consisting of 12 samples with molecular weights ranging from 162 to 6,570,000 g/mol. The copper content in the synthesized nanocomposite was determined on a Shimadzu AA-6200 instrument (Shimadzu Corporation, Kyoto, Japan). The morphology of the nanocomposite and the sizes of CuNPs were determined using a Leo 906E transmission electron microscope (Carl Zeiss AG, Oberkochen, Germany). The zeta potential and hydrodynamic radius of nanoparticles were determined using a ZetaPALS-Zeta Potential Analyser (Brookhaven Instruments Corporation, Holtsville, NY, USA). The measurements were carried out in thermostated cuvettes at a temperature of 25 °C and a scattered light registration angle of 90 °C. Thermooxidative destruction of the polymer and nanocomposite was studied by thermogravimetric analysis and differential scanning calorimetry using an STA 449 Jupiter derivatograph (Netzsch, Selb, Germany). The measurements were carried out in the temperature range of 25–700 °C at a heating rate of 10 °C/min in air. The samples weight was 5 mg.

### 2.5. Preparation of Primary Culture of Rat Hepatocytes

An acute experiment was carried out to isolate the primary culture of hepatocytes of Wistar rats weighing 200 g. The animal experiment was carried out in accordance with the rules of humane treatment of animals. The study was approved by the Ethics Committee of the Irkutsk Scientific Center of Surgery and Traumatology. All surgical manipulations were performed under aseptic conditions. Anesthesia 0.7 mL of a 5% solution of telazol in phosphate-buffered saline (PBS) intramuscularly and chloroform inhalation were used until the loss of locomotor activity but with maintenance of the cardiac muscle contractive activity.

The primary culture of hepatocytes was obtained by perfusion through the portal vein of a solution containing 500 units of heparin in 1 mL of 0.9% NaCl and then a solution of Krebs–Ringer buffer with EDTA without calcium [53] (pH 7.2–7, 4, temperature 37 °C) with a volume of 210 mL with a perfusion rate of 30 mL/min. After that, the buffer was replaced with a 0.07% collagenase solution in HBSS with calcium and magnesium ions (T = 37 °C, 120 mL). Then, the liver was transferred into Hanks’ buffer pre-cooled to the temperature of 4 °C, the liver capsule was opened, the cells were filtered through a cell filter with a pore size of 70 μm and washed in Hanks’ buffer followed by centrifugation at 50× *g* for 5 min at T = 4 °C. Cells were resuspended in William’s E nutrient medium with 10% FBS and Primary Hepatocyte Maintenance Supplements composed of Dexamethasone and Cocktail solution containing GlutaMAX™, HEPES, penicillin-streptomycin, insulin, transferrin, selenium complex, BSA and linoleic acid added to the medium according to the manufacturer’s instructions. The hepatocyte culture was incubated in the 24-well plates at 37 °C, 90% humidity, and 5% CO_2_ in Biostation CT (Nikon Corporation, Tokyo, Japan).

The cells were used for the experiment within 24 h after isolation since no changes in the hepatocyte enzymatic systems functioning were observed during this period [53,54].

### 2.6. Incubation of Rat Hepatocytes with NC(Cu-PVI)

Two hours after hepatocyte isolation, the nutrient medium was replaced and a solution of NC(Cu-PVI) or of CuAc_2_ was added to the culture medium to a final concentration of 50 μg/mL or 200 μg/mL in terms of copper content. A culture of hepatocytes without the addition of test substances served as a control (the culture was supplemented with an appropriate amount of nutrient medium). After 6 and 24 h, the nutrient medium was taken to determine the alanine transaminase (ALT), aspartate transaminase (AST), and alkaline phosphatase (ALP) activity.

### 2.7. Biochemical Research

Biochemical studies of the culture medium after hepatocyte incubation with the test substances were carried out on a Mindray BS-380 biochemical analyzer (Mindray, Shenzhen, China). The activity of ALT, AST was investigated by the kinetic method using the set of reagents for determining the activity of ALT- and AST-UV-Novo liquid form. Alkaline phosphatase was investigated using the Alkaline phosphatase Kit, IFCC Modified Method.

### 2.8. Immunofluorescence Studies

Cells for immunofluorescence studies were fixed with 70% ethanol for 2 h and stained with antibodies to CcO (anti-OxPhos Complex IV subunit I monoclonal antibody) and Anti-Glutathione Synthetase antibody (GS) in a dilution of 1:200. Alexa fluor 568 goat anti-mouse IgG (H + L) or Alexa fluor 488 goat anti-rabbit IgG (H + L) in a dilution of 1:300 was used as secondary antibody. Nuclei were stained with Hoechst, 1:300 [55,56]. The fluorescent stain intensity for each preparation was measured using the Nis-elements software (Nikon Corporation, Tokyo, Japan).

Statistical analysis was carried out in the programming environment R. Single-factor analysis of variance was performed using Kruskal–Wallis test. Post hoc analysis was carried out with applying the Tukey test. Mann–Whitney–Wilcoxon test was used for pairwise comparison.

## 3. Results and Discussion

### 3.1. Characterization of Poly-N-vinylimidazole

**Scheme 1:**
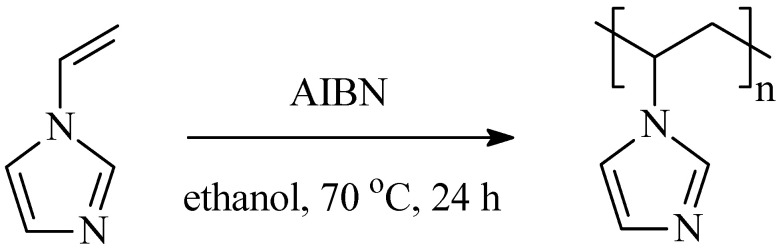
Synthesis of poly-N-vinylimidazole (PVI).

Polymerization of VI in the presence of the AIBN initiator in an ethanol solution and argon atmosphere proceeds according to the free radical mechanism (Scheme 1).

The resulting polymer is a white powder readily soluble in water, DMSO, DMF, and DMAA. For further study of the polymer, a fraction with Mn, Mw and Mw/Mn being 15.635, 20.623 kDa and 1.32, respectively, was isolated by fractional precipitation from an ethanol solution using acetone and hexane as precipitants.

**Figure 1:**
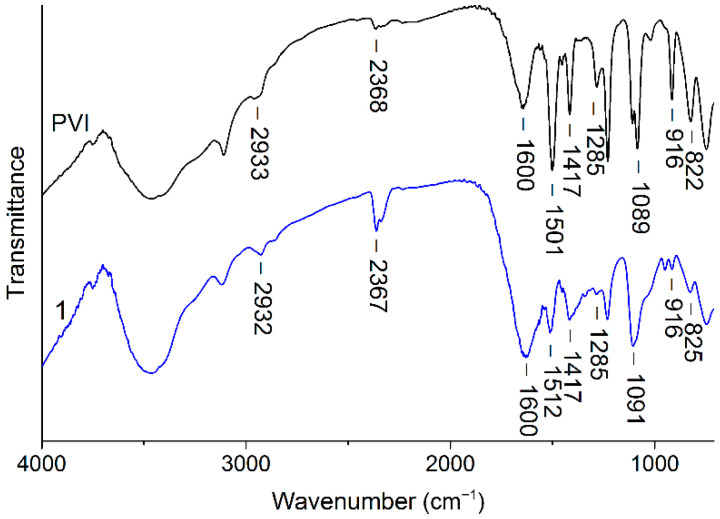
FTIR-spectra of PVI and NC(Cu-PVI) (1).

The FTIR spectra of the polymer contains absorption bands of stretching vibrations of the imidazole fragment: 2933 (C–H), 1501, 1600 (C=N), 1417 (C–N), 1285 (C–H) cm^−1^; CH groups of the azole ring: 1110 and 1089 cm^−1^; and bending vibrations of the heterocycle: 916, 822 cm^−1^ (Figure 1). The band between 3650 and 3300 cm^−1^ refers to the stretching vibration of physically bound water. The ^1^H NMR spectra of PVI contains an intense signal from the protons of the main polymer chain and the imidazole fragment: 1.92–2.34 ppm. (m, 2H, CH_2_), 2.95–3.14 ppm. (m, 1H, CH), 6.65–6.69 ppm. (m, 3H, imidazole ring). PVI is characterized by high resistance to thermal oxidative degradation since the thermal decomposition of the polymer begins above 380 °C.

### 3.2. Synthesis and Characterization of Polymeric CuNPs Nanocomposite

The synthesized PVI is of great importance in the formation of a CuNPs nanocomposite. Active functional imidazole groups in the polymer structure participate in coordination interaction with copper ions ensuring their uniform distribution in the polymer matrix. This occurs at the first stage of the process with stirring of aqueous solutions of PVI and CuAc_2_ and is accompanied by an increase in the viscosity of the solution and the formation of a yellow suspension. With the subsequent portion-wise addition of ascorbic acid to the reaction mixture, copper ions were gradually reduced to zero valence, and the mixture became dark brown. The resulting CuNPs were stabilized in the nanoscale state and retained a uniform distribution over the volume. Ascorbic acid has a significant advantage over sodium borohydride and hydrazine, which are widely used for copper reduction since it is an environmentally friendly and natural antioxidant. The reaction was carried out in an argon atmosphere to prevent possible oxidation of the resulting nanoparticles. The obtained nanocomposites with CuNPs were isolated and purified by dialysis for 48 h followed by freeze drying.

The FTIR spectra of the CuNPs nanocomposite are almost identical to the FTIR spectrum of the PVI, which indicates the stability of the polymer matrix. Only a slight shift (by 11 cm^−1^) of the absorption band of the C=N bond of the imidazole fragment at 1501 cm^−1^ is observed. This change confirms the coordination interaction of copper with the pyridine nitrogen atom (Figure 1).

**Figure 2:**
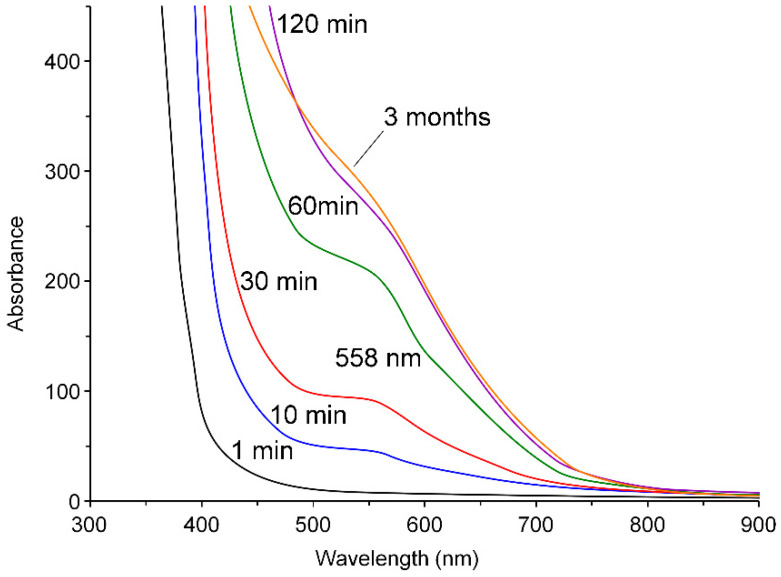
UV–visible spectra of NC(Cu-PVI).

The formation of CuNPs was confirmed using surface plasmon resonance spectra in the UV–visible region. Figure 2 shows the optical absorption spectra of aqueous solutions of the reaction mixture, reflecting the kinetic features of the reaction process. The formation of copper nanoparticles begins after 10 min of the reaction, which is accompanied by the appearance of an absorption band with a maximum at 558 nm, which is characteristic of the plasmon absorption of zero-valent copper nanoparticles. With further stirring of the reaction mixture, the absorption intensity increases and the process of nanocomposite formation is practically completed in 120 min. The position of the plasmon absorption band (588 nm) in the UV spectrum of the synthesized nanocomposite confirms the formation of CuNPs metal nanoparticles. It has been reported in the literature that the surface plasmon resonance band of Cu, CuO and Cu_2_O nanoparticles provides absorption in the UV spectra range 570–600 nm [57], 200–350 nm [58,59,60,61] and 480–485 nm [62,63], respectively.

**Figure 3:**
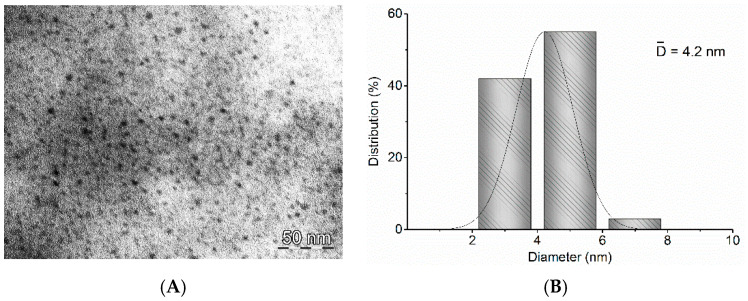
TEM image (**A**) and sizes histogram of NC(Cu-PVI) (**B**).

The use of the method of transmission electron microscopy made it possible to study the size and shape of the formed CuNPs, as well as the nature of their distribution in the PVI polymer matrix. As seen in Figure 3, CuNPs are fairly uniformly distributed in the matrix of the synthesized nanocomposite. Nanoparticles have a spherical shape and are characterized by sizes in the range of 2–8 nm. The predominant amount of nanoparticles (55%) has a size of 4–6 nm. The average nanoparticle diameter is 4.2 nm. Such a small size of nanoparticles and their low content (1.0%) contributed to the fact that the synthesized nanocomposite is readily soluble in water, which is important for the development of biomedical materials.

**Figure 4:**
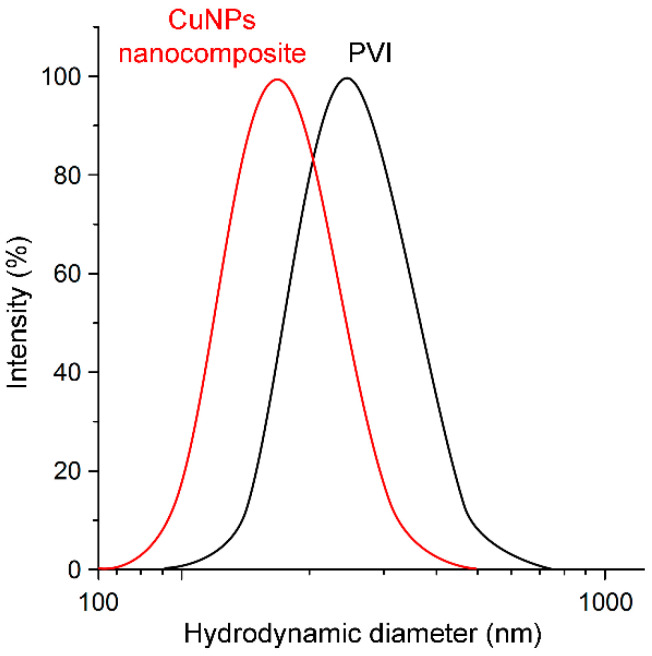
Histogram of the distribution over the hydrodynamic diameters of the scattering particles of NC(Cu-PVI) and the initial PVI.

The average hydrodynamic diameters of the PVI and the nanocomposite are measured by dynamic light scattering. The hydrodynamic diameter of the nanocomposite particles is 166 nm (polydispersity of 0.27), which is 1.46 times smaller than that of PVI (242 nm, polydispersity of 0.22) (Figure 4). This is apparently due to the fact that, during the formation of nanoparticles, their coordination interaction with the pyridine nitrogen atom of the imidazole fragment of the polymer macromolecule leads to compression PVI macrochains.

According to the data of thermogravimetric analysis, the CuNPs nanocomposite shows lower stability to thermal oxidative degradation in comparison with PVI. The onset of thermal decomposition of the nanocomposite is 360 °C, which is 20 °C lower than the thermal stability of PVI. This is probably due to the catalytic properties of metal nanoparticles, which are manifested in a decrease in the activation energy of thermal destruction and oxidation of the polymer matrix.

The obtained nanocomposites are characterized by high stability of the nanoscale state of nanoparticles, which is ensured by the high stabilizing ability of PVI. Even with long-term storage of aqueous solutions of the nanocomposite (more than 3 months), the nanoparticles did not agglomerate, and all the chemical and thermal properties were retained.

### 3.3. Influence of NC(Cu-PVI) on the Morphology of Hepatocytes

In this study, the choice of a cell culture to estimate the cytotoxicity of a copper-containing nanocomposite was dictated by several reasons. First, hepatocyte cultures are widely used in the screening of pharmaceuticals including for ADME/Tox testing (absorption, distribution, metabolism, excretion and toxicity). This is attributed to the ability of liver cells to metabolize endogenous and xenobiotic compounds through the formation of water-soluble components [53,64]. Conversely, the exceptional role of the liver in copper metabolism suggests that hepatocytes are the main target for the toxic effects of this metal and bear the major metabolic load when excess amounts of this trace element intakes to organism [13,14,15,16]. Based on this information, for the research, the primary culture of rat hepatocytes was chosen as the main tool for assessing the toxicity of the new copper-containing nanocomposite for mammalian cells and comparing its action on liver cells with an organic copper salt.

**Figure 5:**
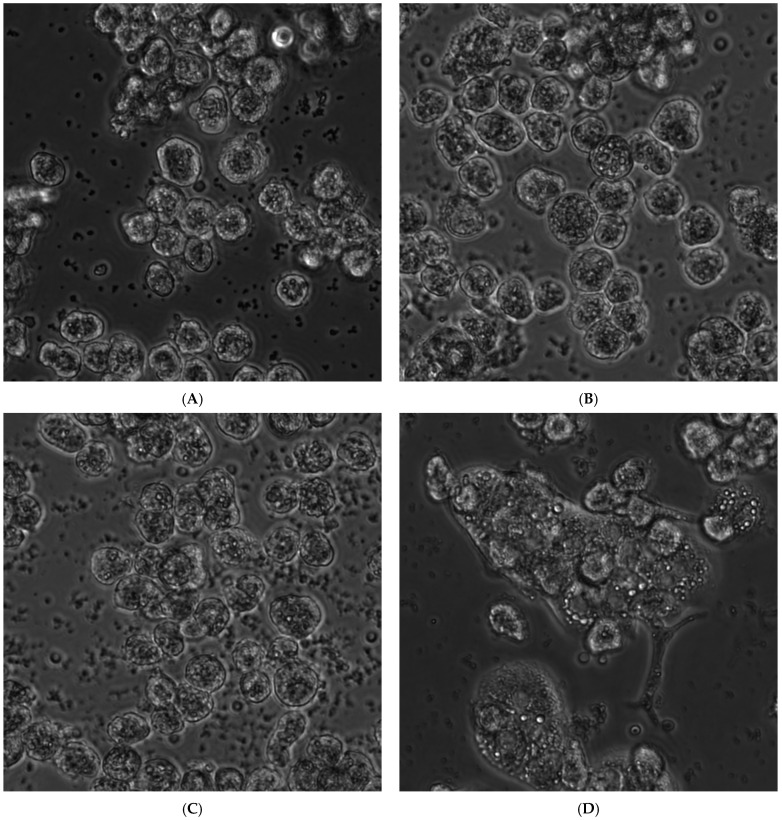

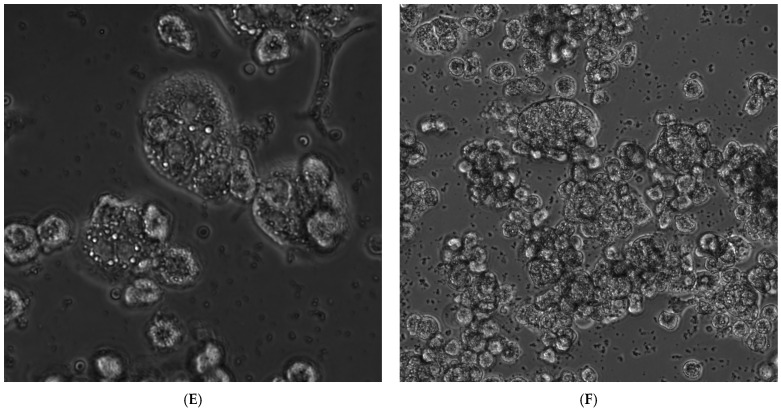
Culture of primary hepatocytes after incubation for 6 h (**A**,**C**,**E**) and 24 h (**B**,**D**,**F**) with NC(Cu-PVI) (**C**,**D**) and CuAc_2_ (**E**,**F**). (**A**,**B**) Control samples. Magnifications are 40× (**A**–**E**), 20× (**F**).

We found that after incubation for 6 h with NC(Cu-PVI), the cultured cells looked intact, and no visible changes in their morphology were found (Figure 5C). However, compared with the control group (Figure 5A), an increase in the amount of cell debris was observed in these samples, which indicates that the processes associated with cell death are enhanced in culture. At the same time, in the samples that were exposed to CuAc_2_, after 6 h of incubation, the formation of cell conglomerates was visualized (Figure 5E), and this process intensified within 24 h that led to the appearance of large areas formed by such conglomerates (Figure 5F). Similar processes were not found in the control samples either after 6 or 24 h of cultivation (Figure 5A,B). However, the long-term exposure to hepatocytes (for 24 h) with the NC(Cu-PVI) also led to the formation of cell conglomerates in culture (Figure 5D). The data obtained indicate that the changes in the cell adhesion properties and the ability to intercellular interactions observed in this experiment are, apparently, initiated by copper preparations, and this process starts earlier and proceeds more intensively in the presence in the medium of this metal ion not bound by the synthesized matrix.

### 3.4. Analysis of Cytotoxicity NC(Cu-PVI) Based on the Activity of AST and ALT

The study of the ALT and AST activity has recently been used, not only in clinical practice to diagnose diseases associated with impaired liver function, but also to research the toxicity of various drugs to 2D and 3D hepatocyte cultures [65,66,67,68]. Based on this, in our investigation, the analysis of the AST and ALT activity in the culture medium was used to determine the degree of copper-containing compound toxicity in relation to the primary culture of hepatocytes.

**Figure 6:**
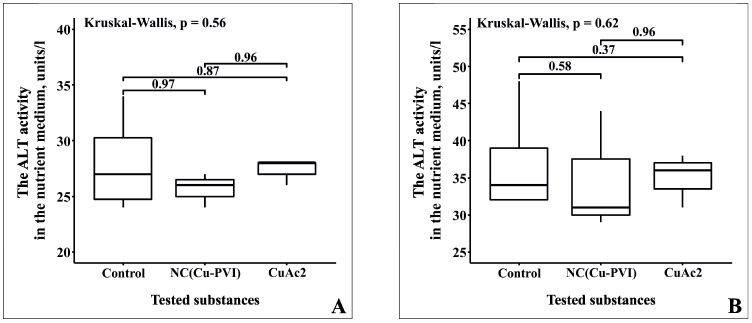

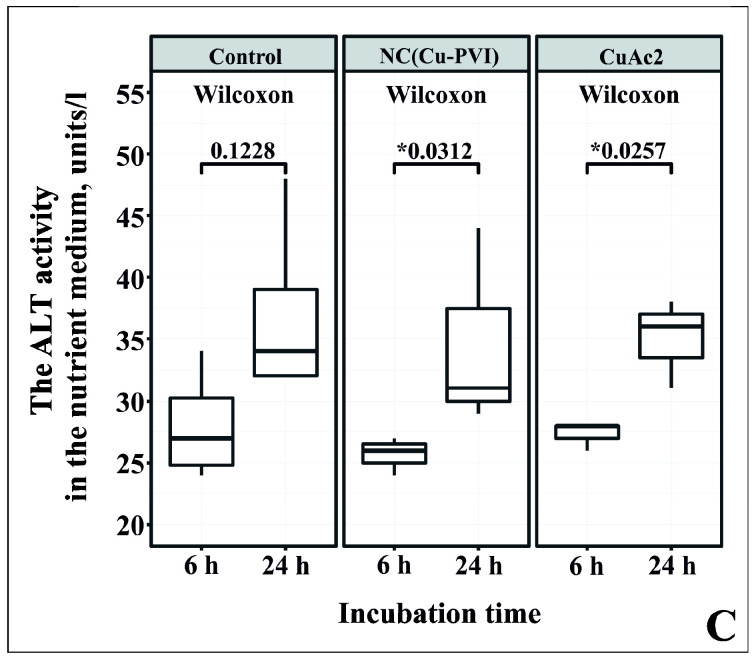
Change in ALT activity in the culture medium after 6 h (**A**) and 24 h (**B**) of incubation with the NC(Cu-PVI) or CuAc_2_ compared with the control group and depending on the duration of incubation (**C**). Medians and first and third quartiles are shown. * Differences are considered significant at *p* < 0.05.

Statistical analysis of the data showed that at the selected time points (6 h and 24 h), the ALT activity in the samples exposed to the NC(Cu-PVI) and CuAc_2_ did not significantly differ from the enzyme activity in the control specimens (Figure 6A,B). Nevertheless, during the experiment, after incubation with both analytes, a significant growth in ALT activity was observed (Figure 6C), and although in this case the activity increase level is comparable for the NC(Cu-PVI), for CuAc_2_, the median value for the latter substance was found to be somewhat higher (Figure 6A–C).

**Figure 7:**
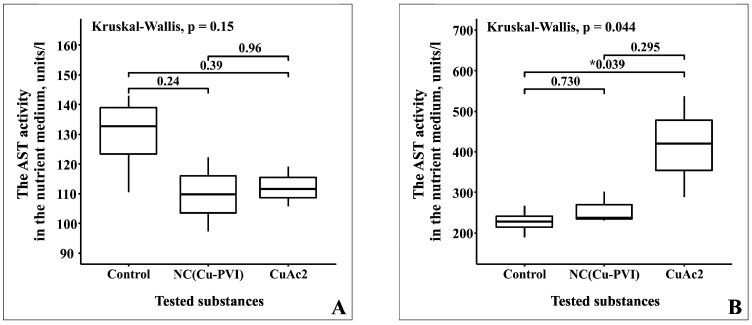

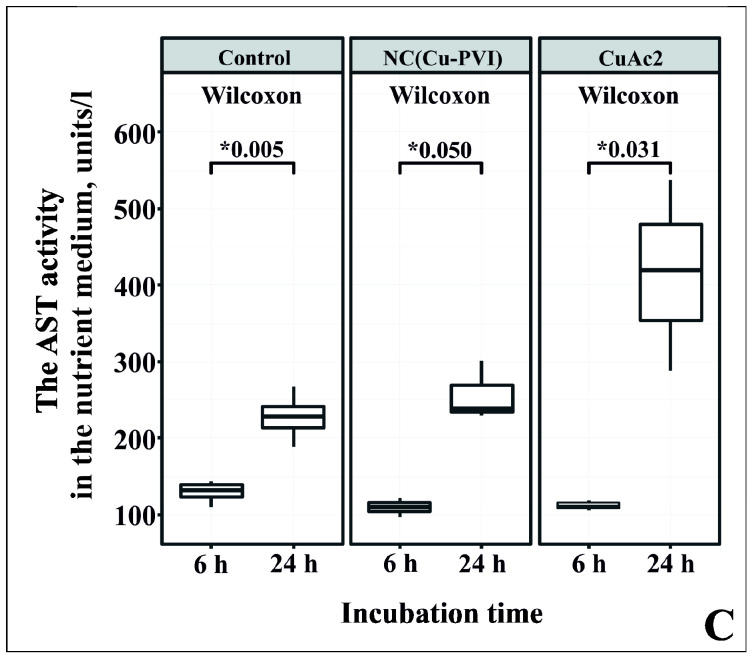
Change in AST activity in the culture medium after 6 h (**A**) and 24 h (**B**) of incubation with NC(Cu-PVI) or CuAc_2_ compared with the control group and depending on the duration of incubation (**C**). Medians and first and third quartiles are shown. * Differences are considered significant at *p* < 0.05.

A similar picture was obtained when determining the activity of AST after 6 h of incubation with copper-containing compounds. The enzyme activity in the experimental samples did not differ significantly from the control group (Figure 7A). However, 24 h after addition of the analytes, the situation has changed. AST activity considerably raised after exposure to CuAc_2_, while incubation with the NC(Cu-PVI) did not lead to a significant increase in enzyme activity (Figure 7B). These data are confirmed by the analysis of AST activity changes recorded at different time points (Figure 7C). As is evident from the graph, in the control samples as well as in the culture incubated with the NC(Cu-PVI), a gradual increase in the enzyme activity occurred, while when exposed to CuAc_2_ for 24 h, a sharp growth in the AST activity was observed in the culture medium that was apparently caused by a significant enhancement in the rate of hepatocyte death in this group of samples.

In our study, ALT and AST, an increase in the activity of which may indicate necrotic death of hepatocytes, demonstrated a different response to treatment of the copper-containing compounds. AST activity significantly distinguished upon incubation of cells with a NC(Cu-PVI) or CuAc_2_, which allows one to estimate the toxic effect degree while ALT activity in this case turned out to be less informative. AST activity growth after exposure to hepatocytes with CuAc_2_ for 24 h is suggested to be caused by the intensification of destructive processes occurring in the cell culture.

In many studies carried out on hepatocyte cultures, an increase in the activity of both aminotransferases in the medium was observed under the influence of various compounds [65,66]. However, in our work, only AST activity growth was demonstrated. Differences in the enzyme activities can be explained by the fact that AST is present in cells in the form of both cytoplasmic and mitochondrial isoenzymes, and the latter form is predominant in hepatocytes [69,70]. It is known that mitochondria play the role of a dynamic pool for copper ions, regulate the cellular homeostasis of this trace element and, accordingly, are the main intracellular targets of the toxic effect of this metal excess amounts [15,71].

Moreover, it is considered that minor damage to hepatocytes leads to the predominant release of enzymes of the cytoplasmic fraction, while severe necrotic lesions are accompanied by the exit of cytosolic and mitochondrial enzymes into the extracellular space [72]. In this regard, an appreciable rise in AST activity and a slight increase in ALT activity (below a significant level) after incubation with CuAc_2_ for 24 h in our study may be associated with the effect of the metal ions on the hepatocyte mitochondria, which results in structural and functional disorders of organelles as well as the development of destructive processes leading to cell death. In the work carried out on a 3D culture of hepatocytes, it was also shown that the release of AST into the culture medium is especially high when cells are exposed to preparations, the toxic effect of which is related with mitochondrial dysfunction [67]. At the same time, our data are consistent with the information that in Wilson–Konovalov’s disease characterized by the accumulation of excess copper amounts in liver cells, a predominant increase in AST activity is recorded in the blood serum, and therefore the AST/ALT ratio exceeding 2 is considered one of the clinical indicators of this pathology [73,74]. In addition, the fact that the appearance of mitochondrial enzymes in blood serum under pathological conditions is usually observed later than cytoplasmic enzymes [75] may explain a significant AST activity growth in the culture medium at later stages of incubation with CuAc_2_.

### 3.5. Effect of NC(Cu-PVI) on ALP Activity

Despite the fact that ALP is not an enzyme localized exclusively in the liver, the determination of its activity is widely used in clinical diagnostics to make or confirm diagnoses associated with pathological processes leading to the degradation of hepatocytes. Based on this, in our study, the determination of this enzyme activity in the culture medium was used as one of the criteria indicating cell damage under the action of a NC(Cu-PVI) and CuAc_2_.

**Figure 8:**
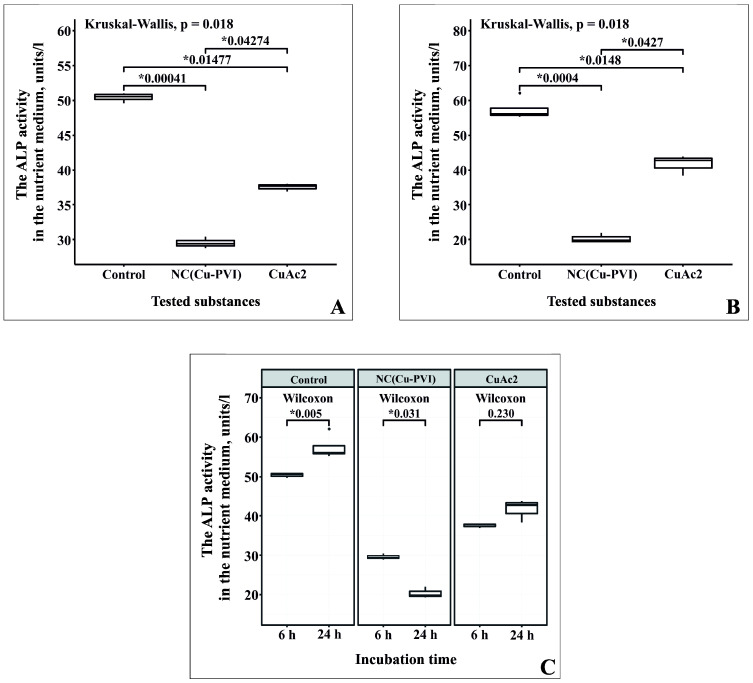
Change in ALP activity in the culture medium after 6 h (**A**) and 24 h (**B**) of incubation with the NC(Cu-PVI) or CuAc_2_ in comparison with the control group and depending on the duration of incubation (**C**). Medians and first and third quartiles are shown. * Differences are considered significant at *p* < 0.05.

Our results showed that, in the culture medium after exposure to the test substances, contrary to expectations, a significant decrease in ALP activity was found in the experimental samples in comparison with the control group. In this case, the enzyme activity reduction was observed both after 6 h and after 24 h of incubation with the tested copper-containing compounds (Figure 8A,B). Moreover, the analysis of the selected time points showed that, after exposure to the nanocomposite, a more intense decrease in ALP activity occurred versus after the addition of CuAc_2_ to the culture medium (Figure 8A,B).

Comparison of the ALP activity in different time points showed that the changes occurring in the cultured cells from the three experimental groups throughout the experiment are of a different nature (Figure 8C). In the control group, a gradual increase in ALP activity in the medium was observed, which is associated with moderate death of hepatocytes in the maintained culture. At the same time, in the samples that were exposed to the NC(Cu-PVI), the activity of ALP continued to considerably decrease over 24 h, whereas after incubation with CuAc_2_, the enzyme activity reduced in the first hours of exposure, but no significant changes were observed in the next 18 h, although ALP activity in this group was still lower than in control samples (Figure 8C).

In this study, the decrease in the activity of ALP in the medium may be associated not with the reduce in the destruction rate in the culture of hepatocytes but with the direct interaction of copper atoms with the analyzed enzyme leading to the suppression of its activity. For example, it was previously shown that copper can displace zinc atoms playing the role of a cofactors in the ALP catalytic centers. This leads to the loss of its functionality and a decrease in the activity recorded by standard methods [76]. Substitution of zinc in the catalytic centers of the enzyme also explains the reduction in the ALP activity level of the blood serum observed in patients with Wilson–Konovalov disease, especially when it manifests itself in the fulminant form accompanied by extensive necrosis of the liver [77,78]. In the case of this disease characterized by the accumulation of excessive amounts of copper in the liver, a decrease the ALP activity in serum does not reflect the degree of hepatocellular damage but is one of the diagnostic signs indicating the toxic effect of copper [73,74,79]. A similar effect of copper preparations on ALP activity was also previously described; however, in contrast to our results, in these studies, only complexes of myo-inositol hexakisphosphate with Cu (II) ions but not free copper ions had an inactivating effect (the enzyme activity decreased by 99%) [80,81]. In our work, both CuAc_2_ and NC(Cu-PVI) had an inhibitory effect. Although copper atoms fixed in the structure of the nanocomposite are not only able to interact with ALP affecting enzyme activity, they also exceed free copper ions in the intensity and duration of ALP inactivation. This may indicate that the synthesized matrix does not prevent the interaction of the metal ions inserted in its structure with enzymes nor does it reduce their bioavailability.

### 3.6. Influence of NC(Cu-PVI) on the Activity of Oxidative Phosphorylation in Hepatocytes

In this study, to assess the effect of the NC(Cu-PVI) on the oxidative phosphorylation intensity of cultivated hepatocytes in comparison with CuAc_2_, cells were stained by the immunofluorescence method using antibodies to subunit I of mitochondrial cytochrome c oxidase (CcO).

**Figure 9:**
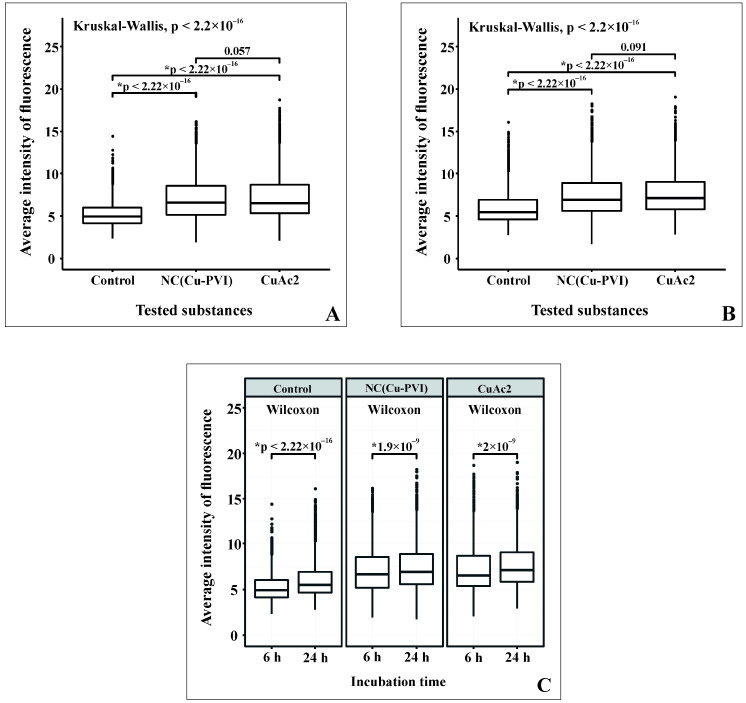
Changes in the cytochrome c oxidase fluorescent stain intensity in the hepatocyte culture after 6 h (**A**) and 24 h (**B**) of incubation with the NC(Cu-PVI) or CuAc_2_ in comparison with the control group and depending on the duration of incubation (**C**). Medians and first and third quartiles are shown. * Differences are considered significant at *p* < 0.05.

Analysis of the intensity of fluorescent staining showed that, after incubation of cells for 6 h with both CuAc_2_ and the NC(Cu-PVI), a significant increase in the amount of the visualized enzyme in hepatocytes was observed (Figure 9A) compared to control samples, which indicates an enhancement in oxidative phosphorylation activity in cells. A similar picture was also obtained when comparing with control samples of cells incubated with the test substances for 24 h (Figure 9B). At the same time, throughout the experiment, there were no significant distinctions in the fluorescence intensity observed in cells that were exposed to the copper salt and the NC(Cu-PVI) (Figure 9A,B). This may indicate that these compounds added to the medium in equal amounts (200 μg/mL) caused the mitochondrial CcO level increase in cultured hepatocytes with the same efficiency.

Comparison of the changes, which takes place in the studied cells depending on the time of exposure to these compounds, made it possible to ascertain that a minor but significant increase in the amount of the enzyme occurs during 24 h in all three analyzed groups (Figure 9C). Apparently, in both experimental groups, the additional factor stimulating the rising in the intensity of oxidative phosphorylation compared with the control group is the introduction of copper ions either in the form of acetate or as part of the nanocomposite.

Three copper atoms included in the catalytic subunits not only provide the enzyme functioning but they are also necessary for the assembly and stability of these subunits since in the absence of copper, proteins are rapidly degraded, which prevents the formation of CcO. In addition, it is known that copper deficiency can lead to a decrease in the expression of both nuclear and mitochondrial subunits of CcO [6,82,83,84], while an excessive non-cytotoxic content of copper in cells causes the opposite effect, namely a CcO expression level increase [6,85]. In this regard, the literature discusses the possibility to regulate the mitochondrial CcO functioning and the oxidative phosphorylation activity in general by introducing chelators or, conversely, additional amounts of copper into the cells, modulating the intensity of cellular metabolism and intracellular processes dependent on energy metabolism. This approach can be effective in the development of drugs targeted to the treatment of oncological diseases (by chelation of Cu) as well as the regulation of cell proliferation and differentiation in regenerative medicine (by addition of Cu) [6,86,87].

In this work, it is visually shown that copper nanoparticles included in the synthesized matrix can participate in the regulation of the CcO expression and/or interact with CcO, providing catalytic centers with available copper atoms such as enzyme assembly factors do, and thus modulating the intensity of energy processes occurring in cells. Moreover, in this case, the samples treated with the nanocomposite demonstrate an increase in the amount of CcO at the same level as unbound metal ions introduced into the nutrient medium in form of CuAc_2_.

### 3.7. Influence of NC(Cu-PVI) on the Activity of Glutathione Synthetase in Hepatocytes

In this work, the effect of the compared copper preparations on the redox system of glutathione in hepatocytes was assessed by the immunofluorescence method using antibodies to glutathione synthetase (GS) being the glutathione synthesis second enzyme responsible for the addition of glycine to γ-glutamylcysteine [88].

**Figure 10:**
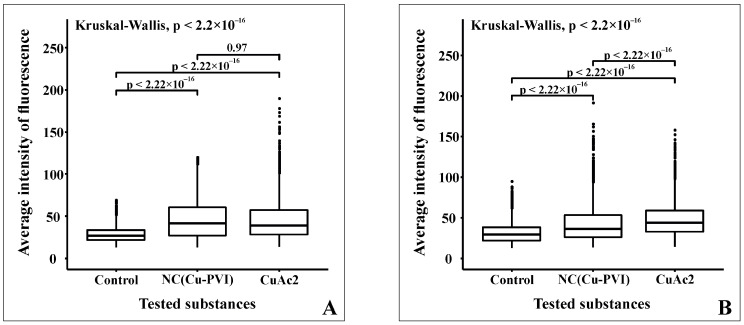

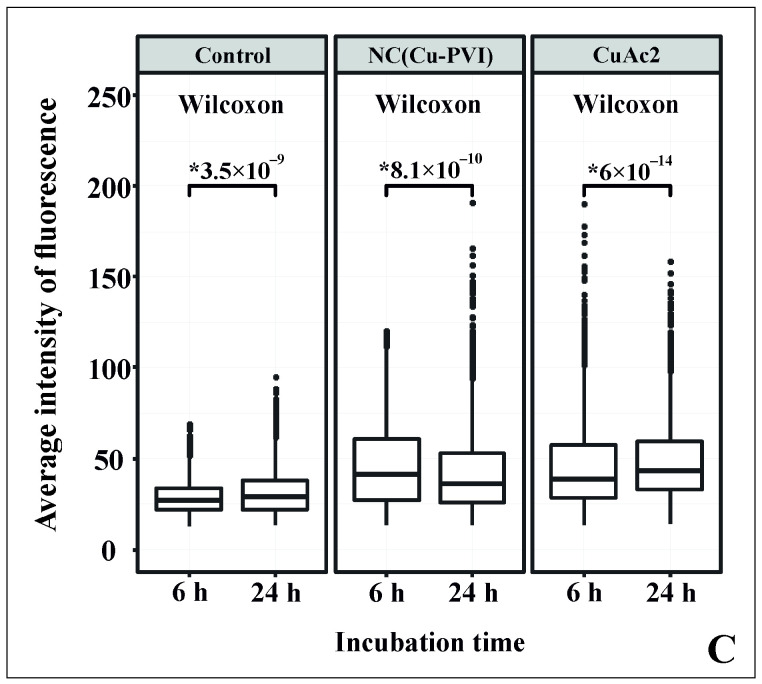
Changes in the glutathione synthetase fluorescent stain intensity in the hepatocyte culture after 6 h (**A**) and 24 h (**B**) of incubation with the NC(Cu-PVI) or CuAc_2_ in comparison with the control group and depending on the duration of incubation (**C**). Medians and first and third quartiles are shown. * Differences are considered significant at *p* < 0.05.

We found that the intensity of staining for glutathione synthetase in cultured cells increases after incubation with both a nanocomposite containing copper and a salt of this metal (Figure 10). As is evident from the graph, after 6 h of exposure to the same concentrations of the tested substances (200 μg/mL), the amount of the enzyme in the cells raises equally without demonstrating significant differences between the effects of the nanocomposite and salt (Figure 10A). However, after 24 h of incubation with these compounds, the situation changes. The fluorescence intensity after exposure to CuAc_2_ becomes significantly higher than in the case of introducing a nanocomposite into the medium (Figure 10C), which indicates that the copper preparation in the form of a salt affects the glutathione synthetase amount in hepatocytes to a greater extent than a nanocomposite in which copper atoms are included in a polymer matrix.

These data are confirmed by comparing the stain intensity depending on the time of exposure to the test compounds (Figure 10C). When hepatocytes are incubated with copper acetate, the amount of the enzyme continues to increase for 24 h, while when exposed to the nanocomposite, growth of the expression level of glutathinone synthetase is observed only in the first 6 h of incubation. In the next 18 h, the amount of the enzyme responsible for the last stage of glutathione synthesis begins to decrease.

Despite the abundance of information on the role of glutathione in maintaining copper homeostasis [5,89,90], there are no data that the ions of this metal can take part in the regulation of this tripeptide synthesis in mammalian cells. Nevertheless, in our study, an increase in the enzyme amount in cultured hepatocytes under the action of two different copper-containing preparations suggests that such regulation takes place. A similar effect has been described in the bacteria *Acidithiobacillus ferrooxidans*, in which, upon exposure to copper, a growth of the expression level of two genes encoding glutathione syntheses was observed [91].

The nature of this regulation remains unclear. It may be related to the direct interaction of copper atoms with metal-sensitive transcription factors, as in the case of metallothioneins, the expression of which is regulated by the metal-responsive transcription factor 1 (MRF-1) interacting with metal-responsive elements (MRE) in gene promoters [2]. Otherwise, it may depend on the glutathione pool depletion in cells and can be realized by a more complicated manner with the involvement of mediator molecules. However, based on our data, it can be assumed that regulation depends on the state of copper atoms present in the cell content, and copper ions in a free form, unbound with the synthesized matrix or cellular proteins, are of crucial importance.

## 4. Conclusions

New water-soluble stable nanocomposites with CuNPs were synthesized based on poly-N-vinylimidazole. The high efficiency of the polymer was found to stabilize CuNPs in a nanosized narrowly dispersed state and ensures the stability of the chemical and thermal properties of the nanocomposite for a long time.

Our investigation showed that the obtained nanocomposite exhibits low toxicity toward cultured rat hepatocytes. It was demonstrated both by cell morphology assay and the ALT and AST activity analysis in culture media. In particular, this nanocomposite was found to be of low toxicity for hepatocyte mitochondria, considering the main intracellular target for the toxic effect of copper ions in excessive amounts. At the same time, it was shown that the chosen matrix does not prevent copper included in the nanocomposite structure to interact with various enzymes, modulating their activity. In this work, it was found that the action of the nanocomposite on cells leads to an increase in the amount of CcO, one of the key enzymes of oxidative phosphorylation. In addition, glutathione synthetase, the second enzyme for the synthesis of glutathione, is involved in chelating the predominant amount of incoming copper in mammalian cells. The opposite effect was observed when analyzing the activity of alkaline phosphatase. In this study, the described effects can be caused by the direct interaction of the nanocomposite with the enzyme, which leads to an increase in the activity of copper-containing CcO or to a more intense and prolonged decrease in the ALP activity associated with the replacement of zinc ions by copper ions in the enzyme active center. At the same time, the effect of the nanocomposite can be explained by its participation in the regulation of the CcO or GS gene expression or, in the case of the latter, by other mechanisms triggered by depleting the glutathione pool. Thus, the synthesized water-soluble copper-containing nanocomposite is promising for biotechnological and biomedical research as a new non-toxic hydrophilic preparation, allowing to regulate the work of key enzymes involved in energy metabolism and antioxidant protection, and it may serve as an additional source of copper.

## Data Availability

Not applicable.
